# Processing of syllable stress is functionally different from phoneme processing and does not profit from literacy acquisition

**DOI:** 10.3389/fpsyg.2014.00530

**Published:** 2014-06-03

**Authors:** Ulrike Schild, Angelika B. C. Becker, Claudia K. Friedrich

**Affiliations:** ^1^Developmental Psychology, University of TübingenTübingen, Germany; ^2^Biological Psychology and Neuropsychology, University of HamburgHamburg, Germany

**Keywords:** spoken word recognition, lexical stress, ERPs

## Abstract

Speech is characterized by phonemes and prosody. Neurocognitive evidence supports the separate processing of each type of information. Therefore, one might suggest individual development of both pathways. In this study, we examine literacy acquisition in middle childhood. Children become aware of the phonemes in speech at that time and refine phoneme processing when they acquire an alphabetic writing system. We test whether an enhanced sensitivity to phonemes in middle childhood extends to other aspects of the speech signal, such as prosody. To investigate prosodic processing, we used stress priming. Spoken stressed and unstressed syllables (primes) preceded spoken German words with stress on the first syllable (targets). We orthogonally varied stress overlap and phoneme overlap between the primes and onsets of the targets. Lexical decisions and Event-Related Potentials (ERPs) for the targets were obtained for pre-reading preschoolers, reading pupils and adults. The behavioral and ERP results were largely comparable across all groups. The fastest responses were observed when the first syllable of the target word shared stress and phonemes with the preceding prime. ERP stress priming and ERP phoneme priming started 200 ms after the target word onset. Bilateral ERP stress priming was characterized by enhanced ERP amplitudes for stress overlap. Left-lateralized ERP phoneme priming replicates previously observed reduced ERP amplitudes for phoneme overlap. Groups differed in the strength of the behavioral phoneme priming and in the late ERP phoneme priming effect. The present results show that enhanced phonological processing in middle childhood is restricted to phonemes and does not extend to prosody. These results are indicative of two parallel processing systems for phonemes and prosody that might follow different developmental trajectories in middle childhood as a function of alphabetic literacy.

## Introduction

Children progressively develop sensitivity to the sound structure of oral language in middle childhood (for review see Goswami and Bryant, 1990; Ziegler and Goswami, 2005). This ability appears to be pivotal for the acquisition of alphabetic writing systems. Children with dyslexia typically have difficulty with detecting or manipulating sounds (e.g., Lyytinen et al., 2004; Ziegler and Goswami, 2005). Once acquired, literacy further shapes phonological awareness. Alphabetic readers outperform illiterate participants in metalinguistic tasks, such as phoneme deletion or phoneme substitution (e.g., Castro-Caldas et al., 1998). The question emerges if progressive refinement of phonological processing in middle childhood is restricted to phonemes or if the processing of speech in general is refined at this age.

Grapheme-to-phoneme correspondence in alphabetic writing systems has been shown to modulate spoken word recognition. Alphabetic readers recognize spoken words more slowly when the words' phonemes can be spelled in different ways than when there is only one spelling for the words' phonemes (Ziegler and Ferrand, 1998). Facilitated word recognition for words with consistent orthography is already evident when normally developing children start reading and writing (Goswami et al., 2005; Ventura et al., 2007, 2008) but is reduced or even absent for children with dyslexia (Zecker, 1991; Desroches et al., 2010). Furthermore, native language orthography appears to have an impact on the processing of non-native language (Mitterer and McQueen, 2009; Escudero and Wanrooij, 2010). Together the findings are captured by the assumption of bi-directional activating links along the pathway of representing and processing spoken language, on the one hand, and written language, on the other (e.g., Grainger and Ferrand, 1996; Grainger and Holcomb, 2009).

Evidence that the development of phonological processing in middle childhood is intimately related to alphabetic literacy comes from functional neuroimaging. By means of fMRI, Brennan et al. (2013) compared neural activation in Chinese and English 8-to-12–year-olds while performing an auditory rhyming task. Rhyming words either were consistent in orthography (e.g., pint-mint) or inconsistent in orthography (e.g., jazz-has). Increased activation of a left-hemispheric phonological network with increasing age, enhanced activation for consistent compared to inconsistent words and a positive correlation between reading skills and superior temporal gyrus activation were found for native English children, but not for native Chinese children. The authors argue that improved phonological awareness and refined phonological processing in English speakers is related to the relatively systematic grapheme-to-phoneme correspondence in English, which contrasts to the relatively arbitrary mapping of written characters to spoken syllables in Chinese.

In line with the assumption of progressively refined phoneme processing as a function of literacy acquisition, we recently found that readers and pre-readers differ in how detailed they process sub-phonemic information in speech recognition (Schild et al., 2011). We tested pre-reading preschoolers, reading preschoolers and second graders by means of the lexical decision latencies and event-related potentials (ERPs) recorded in word onset priming. Spoken syllables (primes) were followed by spoken words (targets). The amount of phoneme overlap between primes and targets was manipulated. For all reading children and for reading adults (Friedrich et al., 2009), a condition in which primes and targets were identical (e.g., in the prime-target pair *mon-Monster “monster”*) differed from a condition in which the onset phoneme of the primes varied in one feature, namely the place of articulation, from the targets (e.g., *non-Monster*). By contrast, “*Monster*” was primed equally well by both primes “*mon*” and “*non*” in pre-reading children. We concluded that readers use more phoneme-relevant detail in lexical access than pre-reading children.

Phonemes are not the only type of information that spoken language entails. Prosody is another source. To establish word prosody, a speaker gives relative emphasis to a certain syllable via enhanced duration, pitch and amplitude. Therewith, phonemically identical syllables might be realized with or without stress. For example, the first syllable of the English word *music* is relatively longer, louder and has higher pitch than the first syllable of the English word *museum*. Similar to written English, written German does usually not code for syllable stress. For example, the stress difference between *August* with stress on the first syllable in spoken German (referring to a male name), and *August* with stress on the second syllable (referring to the month “August”), is not coded in the written forms of those words. For illustration purpose only, we will indicate the stressed syllable of example words by capital letters in the following article (e.g., *MUsic* and *muSEum*, or *AUgust* and *auGUST*).

From a neurocognitive perspective, it appears that the acoustic input is decomposed into phonemes and prosody. Rapidly varying phoneme-relevant information, on the one hand, and more slowly varying prosodic information, on the other hand, are processed by different neuronal networks in adults (Zatorre and Belin, 2001; Boemio et al., 2005; Giraud et al., 2007; Giraud and Poeppel, 2012; Luo and Poeppel, 2012) and in infants (Telkemeyer et al., 2009). In line with this, Event Related Potentials (ERPs) recorded in a previous cross-modal auditory-visual priming study with adults revealed the independent processing of phonemes and pitch contours, as indicated by separate ERP phoneme priming and ERP stress priming (Friedrich et al., 2004).

Previous behavioral priming results show that adults rapidly integrate syllable stress and phonemes in ongoing speech recognition. In cross-modal auditory-visual priming, adults recognize printed words faster when they are preceded by a spoken stress matching syllable, such as the printed word *music* preceded by the spoken stressed syllable *MUS*-, than when they are preceded by a spoken stress mismatching syllable, such as *music* preceded by the spoken unstressed syllable *mus*- (see Cooper et al., 2002 for English; Soto-Faraco et al., 2001 for Spanish; and van Donselaar et al., 2005 for Dutch). Similarly, adults' eye movements are rapidly biased by syllable stress in the visual world paradigm. For example, already before the end of the first syllable of the Dutch word *OCtopus* is encountered, Dutch participants fixate the printed version of *octopus* more frequently than they fixate the printed version of the stress competitor *okTOber* (Reinisch et al., 2010).

In the present study, we focus on the processing of syllable stress in middle childhood. Given the developing phoneme awareness in preschoolers (for review see Goswami and Bryant, 1990; Ziegler and Goswami, 2005) and the refined phoneme processing in beginning readers (Schild et al., 2011), the question emerges whether the processing of all aspects of the speech signal is shaped in middle childhood or whether the refinement of phoneme processing is a function of the acquisition of an alphabetic writing system.

Similar to our previous priming study on the processing of syllable prosody in adults (Friedrich et al., 2004), we orthogonally varied stress-overlap and phoneme-overlap between primes and targets in the present experiment. To make the paradigm appropriate for testing pre-reading children and beginning readers, we had to use a unimodal auditory design in which spoken stressed and unstressed syllables (primes) were followed by spoken disyllabic initially stressed words (targets). This resulted in four prime-target combinations: (i) Stress overlap and phoneme overlap between the prime syllable and the onset of the target word, as in the prime-target pair *MON-MONster*, (“stress-match, phoneme-match”); (ii) Pure stress overlap between the prime syllable and the onset of the target word, as in the prime-target pair *TEP-MONster* (“stress-match, phoneme-mismatch”); (iii) Pure phoneme overlap between the prime syllable and the onset of the target word, as in the prime-target pair *mon-MONster* (“stress-mismatch, phoneme-match”); or (iv) Neither stress nor phoneme overlap between the prime syllable and the onset of the target word, as in the prime-target pair *tep-MONster* (“stress-mismatch, phoneme-mismatch”).

Although unimodal auditory priming has proven to elicit earlier phoneme priming effects than cross-modal priming, other characteristic ERP deflections are largely comparable between both types of paradigms (Friedrich et al., 2009; Schild et al., 2012). Regarding the effects of different onsets of ERPs on unimodal and cross-modal priming, we concluded in our previous studies that phonological processing in the auditory modality is reflected in left-lateralized ERP differences in an early time window, ranging between 100 and 300 ms after the onset of the spoken target word (auditory N100) in adults (Friedrich et al., 2009; Schild et al., 2012) and in infants (Becker et al., 2014; but see Schild et al., 2011 for no effect in children). A left-anterior ERP phoneme priming effect between 300 and 400 ms in both uni- and cross-modal priming, the P350 effect, has been related to matching processes between speech input and lexical representation in adults (e.g., Friedrich, 2005; Friedrich et al., 2009; Schild et al., 2011) and in children (Schild et al., 2012). Finally, an N400-like central negativity starting earlier in unimodal- than in cross-modal priming has been related to predictive phonological processing in adults (e.g., Friedrich, 2005; Friedrich et al., 2008; Schild et al., 2012), in children (Schild et al., 2011) and in infants (Becker et al., 2014). In line with the neurocognitive evidence for independent processing of phoneme-relevant and stress-relevant information (e.g., Boemio et al., 2005; Giraud et al., 2007; Telkemeyer et al., 2009) and based on our previous results (Friedrich et al., 2004), we expect to find independent ERP phoneme priming and ERP stress priming in the present study.

Comparing the processing of syllable stress in pre-readers, beginning readers and adults will enable us to draw conclusions on the middle childhood development of phonological processing. To the best of our knowledge, this study is the first to follow the development of processing of syllable stress over the time related to literacy acquisition in middle childhood. Three possible outcomes could provide insights into language development at that age. First, if the processing of the speech signal in general is refined in readers, they should use syllable stress more effectively than pre-readers. Second, if the processing of speech is refined for those aspects of the speech signal that are relevant in the alphabetic writing system, there might be no difference in how efficiently readers and pre-readers use syllable stress. Third and finally, if literacy draws processing resources away from those aspects of the speech signal that are not coded in the writing system, pre-readers might use syllable stress more efficiently than readers.

## Methods

### Participants

A total of 23 pre-reading preschoolers, 24 beginning readers and 22 adults entered the analysis. Five additional participants were tested but were not included in the final analysis. Two preschoolers did not finish the experiment; and for two beginning readers and for one adult, too few EEG segments remained after artifact correction. Participant characteristics and the results of psychometric tests are summarized in Table 1. All children had normal or above normal IQ scores, as measured with the Raven Colored Progressive Matrices (CPM, Bulheller and Häcker, 2002). In this way we ensured that the differences between groups could not be due to general intelligence. The BISC test (Bielefelder Screening zur Früherkennung von Lese-Rechtsschreibschwierigkeiten, Jansen et al., 2002) indicated that no child was at risk for developing reading or writing impairments. Pre-reading preschoolers were not yet able to read or write words beyond their own name. Beginning readers were at the end of their second year of school. They were able to read at age-appropriate level, as confirmed by a reading test (ELFE1-6, Lenhard and Schneider, 2006). All participants were native speakers of German and were right-handed as assessed by the Edinburgh inventory (Oldfield, 1971). None of the participants reported hearing or neurological problems.

**Table 1:** **Sample size (number of girls/boys and females/males, respectively), age (mean year/month for children and mean years for adults, with respective ranges), mean IQ-score (percentile rank with standard error of mean) accessed with CPM (Bulheller and Häcker, 2002) and handedness (lateralization quotient, LQ, with standard error of mean) accessed by the Oldfield Handedness Questionnaire (Oldfield, 1971) are given**.

	**Sample size**	**Age**	**CPM**	**LQ**
Pre-reading preschoolers	23 (12/11)	6.3 (5.8–6.9)	61.35 (5.12)	87.65 (2.38)
Beginning readers	24 (11/13)	7.11 (7.2–8.11)	66.17 (4.85)	89.83 (2.38)
Adults	22 (5/17)	25 (19–41)	–	85.36 (2.78)

*Pre-reading preschoolers and beginning readers showed no significant differences in CPM or LQ*.

Children were recruited from local schools in Hamburg. Adults were mostly students from the University of Hamburg. They were recruited via mailing lists and internet advertisement. The children and their parents, as well as the adult participants, gave informed consent prior to their inclusion in the study. Children received a gift for their participation (child book or game). The prize of the gift matched the financial compensation of the adult participants. Adults received credit points (students of Psychology) or 8 Euros per hour as compensation for their participation in the study. The study was approved by the Ethics Committee of the German Psychological Association (Deutsche Gesellschaft für Psychologie, DGPs, 10.2006).

### Materials

Forty-five monomorphemic, initially stressed disyllabic German nouns served as stimuli (see Supplementary Material). All of the words had been used in a former study in which we ensured that the words were known by young children (Schild et al., 2011). Pseudowords were created by changing the last phoneme/s of each word (e.g., *Monster* ≥ ^*^Monste).

For the primes, a male native speaker of German (a professional actor) produced the target words once with correctly applied stress (e.g., *MONSter*) and once with incorrectly applied stress (e.g., *monsTER*). We extracted the first syllable of both versions, respectively. Stressed primes were extracted from the correctly stressed version. Unstressed primes were extracted from the incorrectly stressed version. Unstressed primes were realized with full vowels because vowel reduction is not only realized via prosodic parameters but also via the phoneme-relevant parameter vowel quality. In all audio files, the onset of the stimulus was preceded by a 50 ms silent period. The cut-off for the rhymes was the end of the first syllable. If the syllable boundary spanned a plosive speech sound (e.g., MAT-te), the prime was cut after the closure, directly before the release.

**Figure 1:**
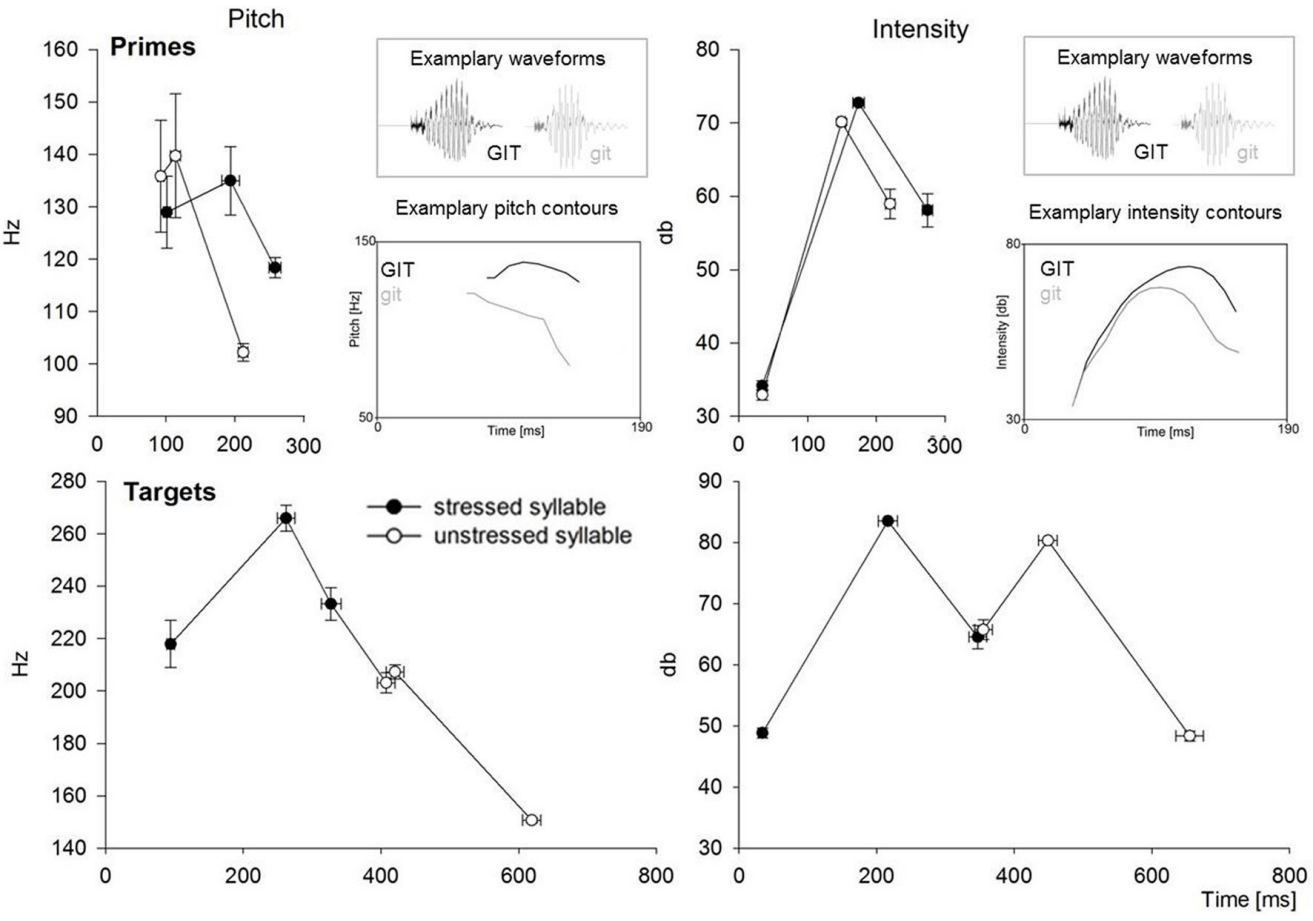
**The figure illustrates the pitch and intensity for the monosyllabic stressed and unstressed primes (above) and the disyllabic initially stressed target words (below) that were presented in the experiment**. Simplified pitch and intensity contours are sketched by the mean first value, the mean maximum value and the mean last value for the monosyllabic primes, as well as for each syllable of the target words. The averaged values are given at the averaged time point they were identified in the signals for stressed and unstressed syllables respectively. Pitch and intensity values were obtained by considering the whole syllable, because the stressed and unstressed syllables were segmentally identical and, therefore, voiced vs. unvoiced segments equally contributed to the pitch contours in both types of syllables. Error-bars indicate standard errors. Measures for stressed syllables are illustrated by black circles. Measures for unstressed syllables are illustrated by white circles. Exemplary intensity and pitch contours for the stressed prime (*GIT* taken from *GITter*, Engl. grid) and the unstressed prime (*git* taken from ^*^gitTER) illustrate the most typical contours. Waveforms of both primes are given for further illustration.

Figure 1 illustrates the realization of syllable stress for the primes (spoken first syllable) and the targets (spoken disyllabic word with initial stress). Amplitude and pitch measures were obtained by analyzing the whole time window of the prime syllables, of the initial syllables of the targets and of the second syllables of the targets, using the software package PRAAT 5.3.17 (Boersma and Weenink, 2014). As is typical, stressed syllables were on average longer and louder than unstressed syllables. Furthermore, stressed syllables showed a pronounced longer period of rising pitch compared to unstressed syllables. This means that the maximum pitch value was reached earlier in unstressed than in stressed syllables. By contrast, the maximum intensity was reached at approximately the same time for stressed and unstressed primes. Therewith, differences in the pitch contours between the stressed and unstressed syllables appear to be earlier available in the signal than differences in the intensity contours.

Targets (words and pseudowords) were spoken by a female native speaker of German (also a professional actor). Digital audio files for each single target were extracted from those utterances. In all audio files, the onset of the stimulus was preceded by a 50 ms silent period. The same target word was presented in four different types of prime-target pairs: (i) Stress overlap and phoneme overlap between prime and target (S+P+, e.g., *MON–MONster*); (ii) stress overlap without phoneme overlap (S+P−; e.g., *TEP–MONster*); (iii) phoneme overlap without stress overlap (S−P+; e.g., *mon–MONster*); and (iv) neither phoneme nor stress overlap (S−P−, e.g., *tep–MONster*). Thus, the stress and phonemes were manipulated independently. The same mapping was applied for pseudowords. To make the task appropriate for children, we had to adapt the lexical decision task, which contained 50% pseudowords, to a go/no-go task, which had only 25% pseudowords. Our pilot testing confirmed that the experiment would have been too long for preschoolers if we had included more pseudoword trials. Moreover, in many priming studies, a lexical decision task is used, in which participants respond to a word with one button and to a pseudoword with another button. Again, our pilot studies showed that these two response alternatives are too demanding for pre-schoolers. Therefore, we decided to use a go/no-go task with a low percentage of non-words (25%) and a single response alternative (“word”).

### Design and procedure

Each participant completed a total of 240 trials (180 target words, 60 target pseudowords). In twelve consecutive blocks, 20 trials were presented each time. Within blocks 1–3, 4–6, 7–9, and 10–12, no repetition of a target word or a pseudoword occurred. Within and across blocks, the order of trials was randomized. In sum, each participant received the same target word four times with four different pairings of primes.

Participants were comfortably seated in an electrically shielded and sound-attenuated booth. Each experimental trial started with the presentation of a “fixation smiley” (size:1 × 1 cm) at the center of a computer screen in front of the participants (distance: 70 cm). Participants were instructed to fixate on this smiley whenever it appeared. The first audio fragment (prime) was presented via loudspeakers 500 ms after the onset of the fixation smiley. The target was delivered 250 ms after offset of the fragment. The interstimulus interval includes the 50 ms silence from the beginning of the wav file for the target. Participants were instructed to respond as quickly and accurately as possible to words but to refrain from responding when the target was a pseudoword (go/no-go task). If an overt response was given, visual feedback (size: 3 × 7 cm) appeared for 2 s. A smiley different from the “fixation smiley” was presented if the participant responded correctly to a word, whereas a ghost was presented if the participant responded to a pseudoword incorrectly. If no response occurred, no feedback was delivered, and the fixation smiley remained for 3.5 s. The next trial started after a 1.5 s inter-trial interval. The loudspeakers were placed on the left and right sides of the screen. Half of the participants pressed the response button with their left index finger, and half, with their right index finger. Auditory stimuli were presented at comfortable listening sound levels of approximately 70 db. Stimulus presentation was controlled by Presentation® software (Version 14.9, Neurobehavioral Systems, Berkeley, CA, U.S.A.).

### EEG-recording and analysis

The continuous EEG was recorded at a 500 Hz sampling rate (bandpass filter 0.01–100 Hz, BrainAmp Standard, Brain Products, Gilching, Germany) from 46 active Ag/AgCl electrodes mounted in an elastic cap (Electro Cap International, Inc.) according to the international 10–20 system (two additional electrodes below the eyes, ground at position AF3). For adults, we recorded from 73 electrodes. After recording with a nose electrode reference, the continuous EEG was off-line re-referenced to an average reference and highpass-filtered by 0.3 Hz.

Eye artifacts were corrected using surrogate Multiple Source Eye Correction (MSEC) by Berg and Scherg (1994), as implemented in the Besa Research-Software® (Version 5.3, MEGIS Software GmbH; Gräfelfing, Germany). Here, brain activity is modeled by a fixed dipole model (the “surrogate model”), and spatial artifact topographies are used to correct the artifacts in the ERP data. To adjust typical artifact topographies to the individual artifact topographies, calibration trials for blinks, vertical and horizontal eye movements were recorded prior to the experiment from the children. The continuous EEG was then corrected for those eye movements by means of a principal component analysis (for details see Berg and Scherg, 1994). Because adults barely moved their eyes in the experiment, for them, only blinks out of the experiment were used and corrected. The remaining artifacts, such as slow drift or movement artifacts, were eliminated according to visual inspection. Individual electrodes showing artifacts that were not reflected in the remaining electrodes in more than two trials were interpolated for all trials. This practice resulted in approximately 2 interpolated electrodes per participant (mean = 2.3, Standard Error of mean [*SE*] = 0.2; not significantly different between groups, all *t* < 1.8, ns).

ERP segments were computed for the target words with correct responses, starting from the beginning of the speech signal up to 1000 ms post-onset of the stimulus and having a 200 ms prestimulus baseline. All data sets included at least 19 segments in each condition (mean/SE across groups: S+P+: 35.2/0.8; S+P−: 35.4/0.7; S−P+: 36.0/0.8; S−P−: 35.2/0.8). There were no significant differences in the numbers of segments in each condition.

### Data analysis

As in our previous study (Schild et al., 2011), responses shorter than 200 ms and longer than 2000 ms, which is approximately in the 2-standard-deviation margin, were removed from the behavioral analyses. Reaction times calculated from the onset of the words up to the participants' responses were subjected to a two-way repeated measures ANOVA with the within-participant two-level factor *Stress Overlap* (prime and target onset match vs. mismatch in stress) and *Phoneme Overlap* (prime and target onset match vs. mismatch in phonemes) and the between-participant three-level factor *Group*.

Because the ERP variance for processing different words is high, targets usually are presented several times in ERP studies so that they are heard in all possible prime-target combinations by a single participant. Consequently, target words were repeated four times in the present experiment. This procedure diverges from classical psycholinguistic designs, in which target repetitions within participants are avoided. To compare the present behavioral results with those of former studies using the classical procedure without target word repetition (Soto-Faraco et al., 2001; Cooper et al., 2002 for Spanish, van Donselaar et al., 2005), we analyzed the first presentation of each target word in addition to the analysis of all presentations.

To analyze the ERP effects, two additional factors were used, *Hemisphere* (left vs. right electrode sites) and *Region* (anterior vs. posterior electrode sites). We calculated the same ROIs as in our former study, namely four lateral ROIs (anterior left: F9, F7, F3, FT9, FT7, FC5, FC1, T7, C5; anterior right: F10, F8, F4, FT10, FT8, FC6, FC2, T8, C6; posterior left: C3, TP9, TP7, CP5, CP1, P7, P3, PO9, O1; posterior right: C4, TP10, TP8, CP6, CP2, P8, P4, PO10, O2) and two central ROIs (anterior: FPz, AFz, Fz, FCz; posterior: Cz, Pz, POz, Iz). In case of significant interactions, *t*-tests were computed to evaluate the differences among conditions. ERP analysis was based on average references. For ERP analysis, only interactions including the factor *Stress Overlap*, the factor *Phoneme Overlap* or both factors are reported. Data analysis was performed with SPSS® software (Version 19, IBM®).

## Results

**Figure 2:**
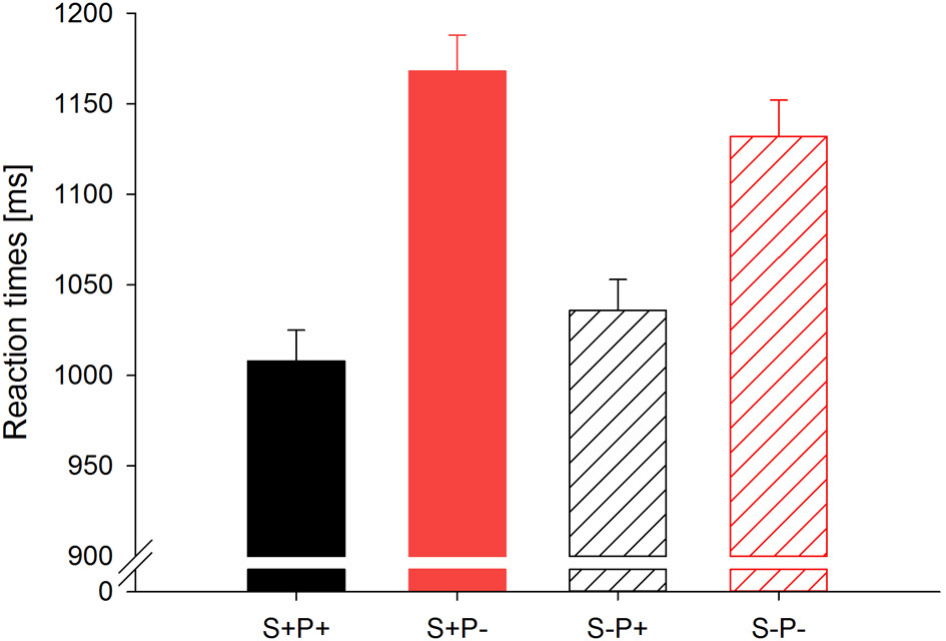
**Mean reaction times across all groups for each condition for the first 60 trials (trials without repetition of the target word)**. Error bars indicate standard errors. The abbreviations of the four conditions are as follows: “S+P+” for stress match, phoneme match (e.g., *MON–MONster*); “S+P−” for stress match, phoneme mismatch (e.g., *TEP–MONster*); “S−P+” for stress mismatch, phoneme match (e.g., *mon–MONster*); and “S−P−” for stress mismatch, phoneme mismatch (e.g., *tep–MONster*). All conditions were significantly different from each other.

The mean reaction times for each group and conditions for the first presentation and overall are given in Table 2, and illustrated for the first presentation in Figure 2.

**Table 2:** **Mean reaction times in milliseconds (and standard error of mean) are shown for each group and each condition**.

	**First 60 trials (no target repetition)**				**All trials (four target repetitions)**			
	**S+P+**	**S+P−**	**S−P+**	**S−P−**	**S+P+**	**S+P−**	**S−P+**	**S−P−**
Pre-reading children	1093 (29)	1295 (25)	1149 (30)	1275 (33)	1082 (27)	1199 (23)	1104 (23)	1176 (27)
Beginning readers	1049 (25)	1228 (27)	1068 (22)	1152 (25)	1083 (23)	1177 (25)	1083 (22)	1154 (27)
Adults	904 (24)	1022 (21)	944 (21)	986 (20)	911 (22)	943 (21)	916 (20)	931 (19)
**Combined groups**	**1018 (18)**	**1185 (20)**	**1056 (17)**	**1140 (21)**	**1028 (17)**	**1110 (19)**	**1037 (16)**	**1090 (19)**

*The results for the first target presentation (without target repetition) are shown in the left columns. The results for all trials (with four target repetitions) are shown in the right columns. Abbreviations for the four conditions are as follows: “S+P+” for stress match, phoneme match (e.g., MON–MONster); “S+P−” for stress match, phoneme mismatch (e.g., TEP–MONster); “S−P+” for stress mismatch, phoneme match (e.g., mon–MONster); and “S−P−” for stress mismatch, phoneme mismatch (e.g., tep–MONster)*.

### Reaction times for the first presentation of the target words

The ANOVA for the first presentation revealed a main effect of the factor *Group*, *F*_(2, 66)_ = 26.2, *p* < 0.001, a main effect of the factor *Phoneme Overlap, F*_(1, 66)_ = 247.4, *p* < 0.001, and an interaction between the factors *Phoneme Overlap* and *Group*, *F*_(2, 66)_ = 9.5, *p* < 0.001. Crucially, there was an interaction between the factors *Phoneme Overlap* and *Stress Overlap*, *F*_(1, 66)_ = 33.2, *p* < 0.001.

The main effect of the factor *Group* indicated that adults responded faster than children. The main effect of the factor *Phoneme Overlap* indicated that all participants responded faster when primes and target onsets shared phonemes than when they shared no phonemes. Follow-ups of the interaction of the factos *Phoneme Overlap* and *Group* indicated that the factor *Phoneme Overlap* was significant for each group, all *F* ≥ 74.2, *p* < 0.001. The mean difference for phoneme match and phoneme mismatch was 79 ms for the adults, 164 ms for the preschoolers and 130 ms for the second graders. Both groups of children showed stronger phoneme priming effects than adults, *F* ≥ 10.1, *p* < 0.01. However, the groups of children did not differ significantly from each other, *F* < 2.4, ns.

Following up the interaction of the factors *Phoneme Overlap* and *Stress Overlap, post-hoc* comparisons indicated that all single conditions differed significantly from each other, all *t*_(68)_ ≥ 4.3, *p* ≤ 0.001. The fastest responses were made when the prime and target onset shared stress and phonemes (S+P+), whereas slowest responses were made when the prime and target onset shared stress but differed in phonemes (S+P−).

### Reaction times overall (four repetitions of the target words)

The ANOVA over all four repetitions of the targets yielded similar results as the ANOVA for the first presentation of the target words; namely, a main effect of the factor *Group*, *F*_(2, 66)_ = 26.6, *p* < 0.001, a main effect of the factor *Phoneme Overlap, F*_(1, 66)_ = 290.3, *p* < 0.001, and an interaction of the factors *Phoneme Overlap* and *Group*, *F*_(2, 66)_ = 30.5, *p* < 0.001, were observed. Again, there was an interaction of the factors *Phoneme Overlap* and *Stress Overlap*, *F*_(1, 66)_ = 12.2, *p* < 0.01.

Similar to the results for the first presentation, the main effect of the factor *Group* over all blocks indicated that adults responded faster than children. The main effect of the factor *Phoneme Overlap* indicated that all participants responded faster when the primes and target onsets shared phonemes than when they shared no phonemes. Follow-ups of the interaction of the factos *Phoneme Overlap* and *Group* indicated that there was a significant phoneme priming effect for each group, all *F* ≥ 26.7, *p* < 0.001. Both groups of children showed stronger phoneme priming than adults, *F* ≥ 41.0, *p* < 0.001. The groups of children did not differ from each other, *F* < 1.3, n.s. The mean difference for phoneme-matching and phoneme-mismatching was 24 ms for adults, 95 ms for preschoolers and 83 ms for second graders.

Again, follow-ups of the interaction of the factors *Phoneme Overlap and Stress Overlap* indicated fastest responses when the prime and target onset shared stress and phonemes (S+P+). The slowest responses were obtained when the targets' first syllables shared stress but differed in the phonemes from their preceding primes (S+P−). *Post-hoc* comparisons revealed significant differences among all conditions, *t*_(68)_ ≥ 3.4, *p* ≤ 0.001, except in the case of targets that shared phonemes but did or did not diverge in stress from their preceding primes (S+P+ vs. S−P+), which was significant at the trend level only, *t*_(68)_ = 1.91, *p* = 0.067.

### Event-related potentials

**Figure 3:**
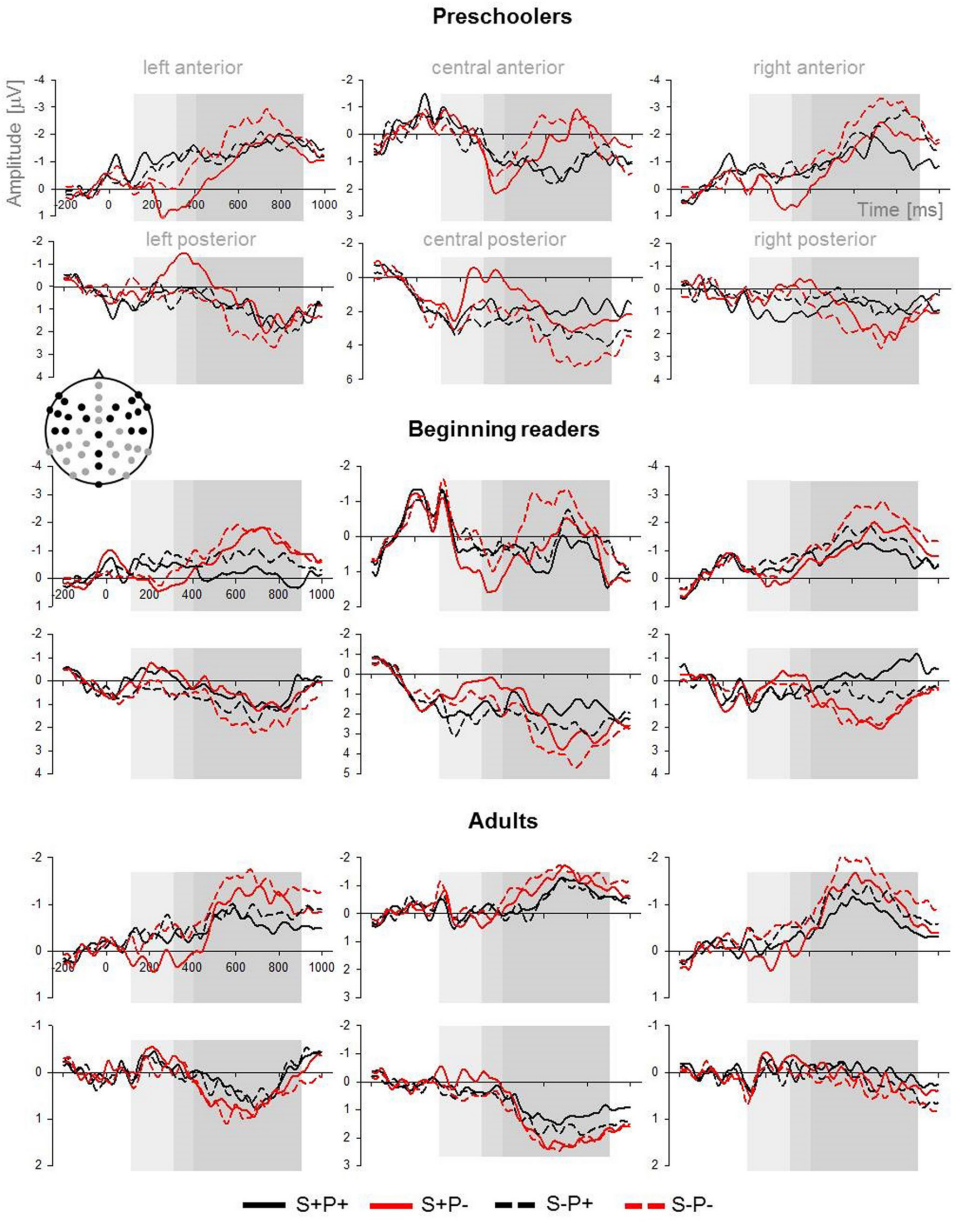
**Mean ERPs over the lateral (left and right) and central and over the anterior and posterior ROIs for each group**. Black and gray dots on the head montage indicate the electrode positions that contributed to the ROIs. The three time windows analyzed in greater detail are highlighted in gray.

**Figure 4:**
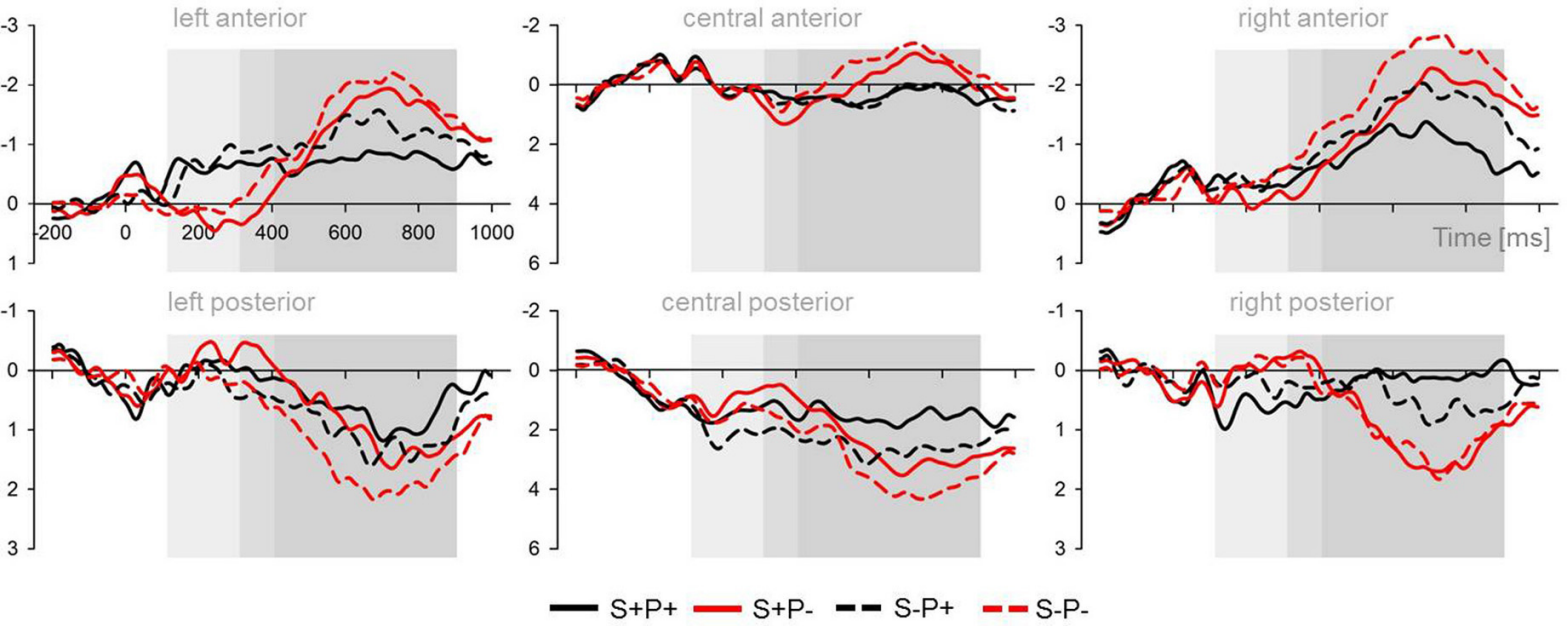
**Mean ERPs over the lateral (left and right panel) and central (middle panel) and over the anterior and posterior ROIs across all groups**. The three analyzed time windows are highlighted in gray.

**Figure 5:**
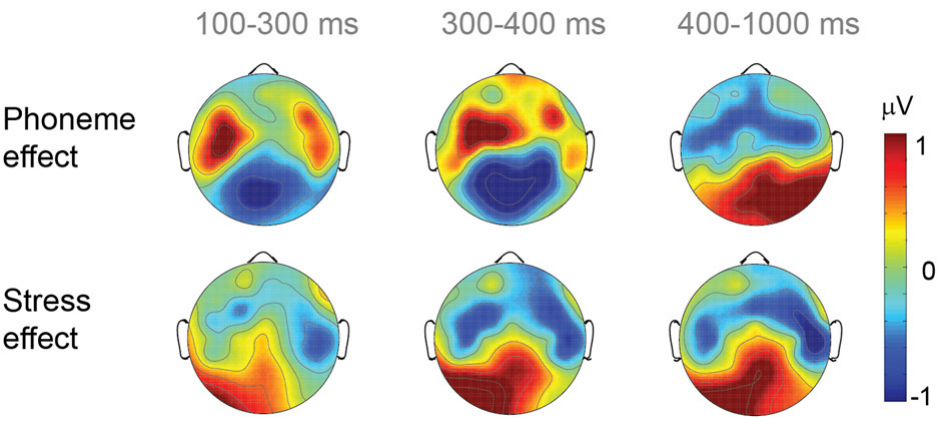
**Topographical voltage maps of the ERP phoneme-priming effect and stress-priming effect (match subtracted from mismatch, respectively) across all groups for the three analyzed time windows**.

The mean ERPs for each of the three groups are displayed in Figure 3. The mean ERPs across all groups for the four ROIs can be seen in Figure 4. We collapsed the ERP over the groups because, in the time windows from 100 to 400 ms, no group effects were observed. For topographical voltage maps of the phoneme and stress-priming effects, see Figure 5. According to consecutive 100-ms time window analyses (see Supplementary Material) and according to previous auditory priming studies (Friedrich et al., 2009; Schild et al., 2011, 2012), we tested the mean ERP amplitudes in three time windows in detail: (i) a time window ranging between 100 and 300 ms addressing auditory phonological processing (N100); (ii) a time window ranging between 300 and 400 ms addressing abstract lexical processing (P350) and predictive phonological processing (central negativity); and (iii) a time window ranging from 400 to 1000 ms capturing extended ERP phoneme priming and ERP stress priming.

#### Time window 100−300 ms (auditory N100)

***Lateral Electrodes***. The overall ANOVA of the lateral ROIs revealed interactions of the factor *Phoneme Overlap* with the factor *Hemisphere, F*_(1, 66)_ = 3.7, *p* = 0.05 and with the factor *Region, F*_(1, 66)_ = 8.4, *p* = 0.005. The overall ANOVA of the lateral ROIs also revealed an interaction between the factors *Stress Overlap* and *Region*, *F*_(1, 66)_ = 4.9, *p* = 0.03.

Follow-ups revealed main effects of the factor *Phoneme Overlap* over the left hemisphere, *t*_(68)_ = 3.4, *p* = 0.001, and over anterior regions, *t*_(68)_ = 3.9, *p* < 0.001. Prime-target pairs matching in phonemes elicited more negative amplitudes than prime-target pairs mismatching in phonemes. There was no significant difference between both conditions over the right hemisphere, and a trend for reversed amplitude differences between conditions over posterior regions, *t*_(68)_ = 1.9, *p* = 0.06. Furthermore, follow-ups revealed a main effect of the factor *Stress Overlap* over posterior regions, *t*_(68)_ = 3.1, *p* = 0.003. Amplitudes for stress match were more negative than amplitudes for stress mismatch. There was no main effect of stress over anterior regions.

***Central Electrodes***. The overall ANOVA of the central ROIs revealed an interaction between the factors *Phoneme Overlap* and *Region*, *F*_(1, 66)_ = 4.6, *p* = 0.04, indicating an effect for the posterior ROI that showed the same amplitude difference as was obtained for posterior lateral ROIs, *t*_(68)_ = 3.5, *p* = 0.001.

Neither over lateral ROIs nor over midline ROIs were any interactions between the factors *Stress Overlap* and *Phoneme Overlap* observed in the first time window.

#### Time window 300−400 ms (P350 and central negativity)

**Figure 6:**
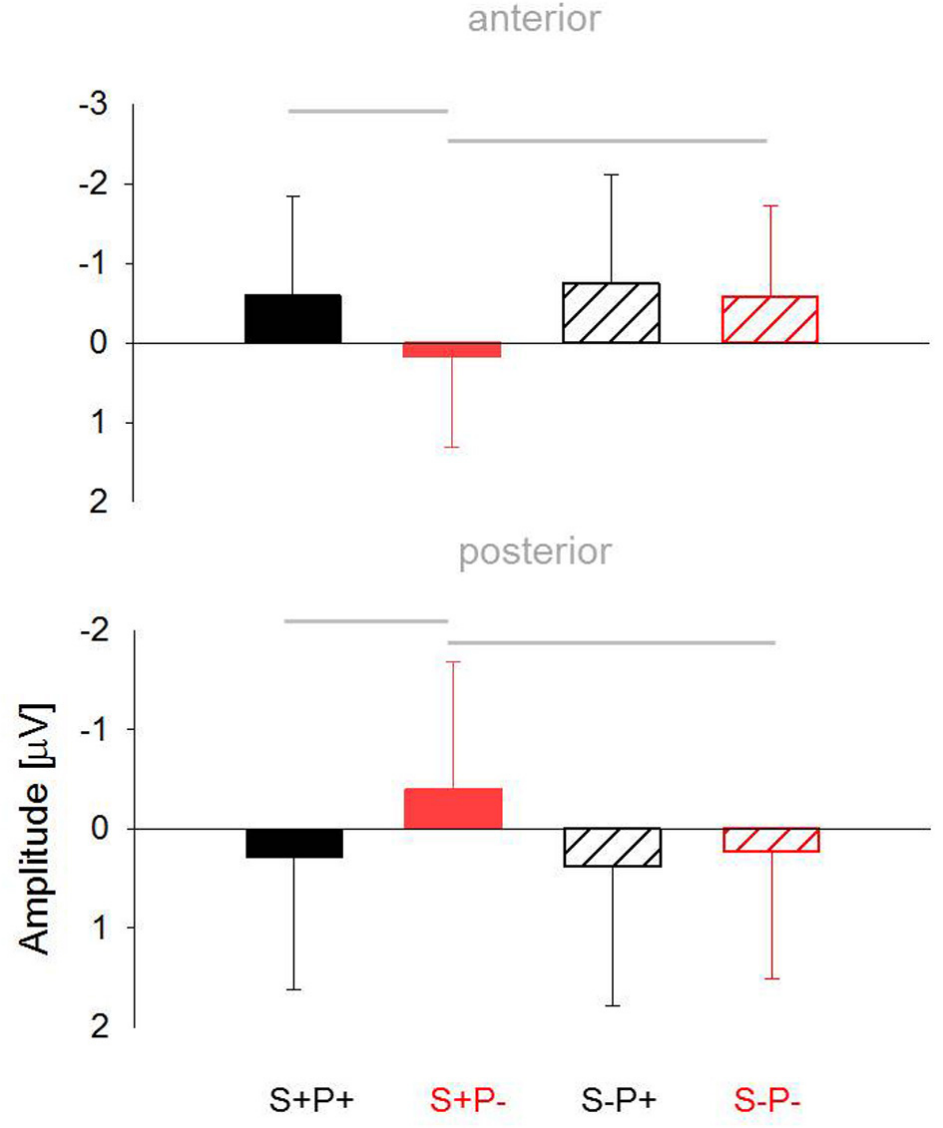
**Mean ERP-amplitudes between 300 and 400 ms elicited for the anterior (upper panel) and posterior (lower panel) ROIs across all groups**. Gray lines indicate significant differences between conditions, as revealed by *post-hoc t*-tests.

***Lateral Electrodes***. In this time window, we found an interaction of the factors *Phoneme Overlap*, *Stress Overlap* and *Region*, *F*_(1, 66)_ = 4.1, *p* = 0.05, for the lateral electrodes. Follow-up analysis revealed a significant interaction between the factors *Phoneme Overlap* and *Stress Overlap* for the anterior regions, *F*_(1, 68)_ = 4.5, *p* = 0.04, and a trend level effect for the posterior regions, *F*_(1, 68)_ = 3.4, *p* = 0.07. Both interactions are illustrated in Figure 6. It appeared that the condition (S+P−) showing the slowest behavioral responses differed in ERP amplitudes from all other conditions, all *t*_(68)_ ≥ 3.5, all *p* ≤ 0.001. All remaining conditions did not differ from one other *t*_(68)_ < 1.1, ns.

***Central Electrodes***. The overall ANOVA of the central ROIs revealed an interaction between the factors *Phoneme Overlap* and *Region*, *F*_(1, 66)_ = 24.0, *p* < 0.001, and an interaction between the factors *Stress Overlap* and *Region*, *F*_(1, 66)_ = 17.5, *p* < 0.001. Follow-ups of effects of the factor *Phoneme Overlap* revealed significantly more negative amplitudes for matching compared to mismatching phonemes over the anterior midline, *t*_(68)_ = 3.0, *p* = 0.004. This pattern was reversed over the posterior midline, *t*_(68)_ = 4.4, *p* < 0.001. Follow-ups of effects of the factor *Stress Overlap* revealed that *s*tress-matching conditions elicited more negative amplitudes than stress-mismatching conditions over the posterior regions, *t*_(68)_ = 4.3, *p* < 0.001.

#### Time window 400−1000 (extended processing)

***Lateral Electrodes***. The overall ANOVA of the lateral ROIs revealed significant interactions of the factor *Phoneme Overlap* with the factor *Region*, *F*_(1, 66)_ = 23.1, *p* < 0.001, and with the factors *Hemisphere* and *Region*, *F*_(1, 66)_ = 5.7, *p* = 0.02. Both interactions were modulated by a four-way interaction of the factors *Hemisphere, Region, Phoneme Overlap* and *Group, F*_(2, 66)_ = 3.2, *p* = 0.05. The overall ANOVA of the lateral ROIs also revealed a significant interaction of the factors *Stress Overlap* and *Region, F*_(1, 66)_ = 23.1, *p* < 0.001.

Follow-up ANOVAS for each group separately revealed that only the preschoolers showed a three-way interaction of the factors *Phoneme Overlap, Hemisphere* and *Region*, *F*_(1, 22)_ = 7.0, *p* = 0.02. Over right posterior regions, phoneme-matching conditions elicited more negative amplitudes than phoneme-mismatching conditions, *t*_(22)_ = 2.4, *p* = 0.03. Both reading groups, the beginning readers and the adults, showed interactions of *Phoneme Overlap* and *Region, both F* > 20.0, *p* < 0.001. For both groups, prime-target pairs mismatching in phonemes elicited more negative amplitudes than prime-target pairs matching in phonemes over anterior regions. The reversed pattern was obtained over posterior regions, all *t* > 3.9, *p* ≤ 0.01.

Follow-ups of effects of the factor *Stress Overlap* revealed that over anterior regions, the amplitudes of the stress-mismatching conditions were more negative than the amplitudes of the stress-matching conditions, *t*_(68)_ = 5.5, *p* < 0.001. This effect was reversed over posterior regions, *t*_(68)_ = 3.7, *p* < 0.001.

***Central Electrodes***. The overall ANOVA of the central ROIs revealed a significant interaction of the factors *Phoneme Overlap* and *Region*, *F*_(1, 66)_ = 30.0, *p* < 0.001, which was not modulated by the factor group. Furthermore, there was a significant interaction of the factors *Stress Overlap* and *Region, F*_(1, 66)_ = 21.6, *p* < 0.001.

Follow-ups of *Phoneme Overlap* effects revealed that the amplitudes of phoneme-mismatching conditions were more negative over the anterior midline than the amplitudes of phoneme-matching conditions, *t*_(68)_ = 3.8, *p* < 0.001. The effect was reversed over the posterior midline, *t*_(66)_ = 3.5, *p* ≤ 0.001. Follow-ups of *Stress Overlap* effects revealed the same amplitude differences as for the lateral ROIs over both the anterior midline, *t*_(68)_ = 1.86, *p* = 0.07, and over the posterior midline, *t*_(68)_ = 4.7, *p* < 0.001.

Neither over lateral ROIs nor over midline ROIs were any interactions between the factors *Stress Overlap* and *Phoneme Overlap* observed in the third time window.

In summary, the ERP data were quite comparable for preschoolers, beginning readers and adults. For all groups, phoneme priming started at approximately 100 ms, and stress priming started at approximately 200 ms (see Supplementary Material). Across all three larger time windows, the ERPs of all groups showed independent ERP priming effects for prime-target overlap in phonemes, on the one hand, and for prime-target overlap in stress, on the other hand. ERP phoneme priming was characterized by enhanced N100 for phoneme match and enhanced P350 and central negativity for phoneme mismatch. ERP stress priming was characterized by sustained enhanced negativity for stress match. Only in the time window ranging between 300 and 400 ms did phoneme priming and stress priming interact over lateral electrodes. Nevertheless, even in this time window, independent phoneme priming and stress priming was obtained over the midline electrodes.

## Discussion

The present study focused on the processing of syllable stress in middle childhood. We tested pre-readers and beginning readers, as well as adults. Behavioral and ERP stress priming were comparable across groups. Thus, we can discard the first hypothesis stating that the processing of the speech signal in general is improved in readers, and also the third hypothesis stating that the readers withdraw processing resources from aspects of the speech signal that have no correspondence with the writing system. Instead, adults, pre-readers and alphabetic readers appeared to similarly exploit syllable stress. Together the present results speak for the second hypothesis, stating that alphabetic readers' sensitivity is not enhanced regarding an aspect of the speech signal that does not correspond with the writing system, namely syllable stress.

The group effects in the present data suggest that refined speech processing in middle childhood is restricted to phonemes. The behavioral data indicate stronger phoneme priming effects, but not stronger stress priming effects, in children compared to adults. The ERPs point to a unique late ERP response to phoneme priming for preschoolers, but stress priming does not show a unique ERP response for any group. Together, these results reveal that, in middle childhood and especially at the preschool ages, phonological awareness might drive portions of the phoneme priming effects. That is, preschoolers and beginning readers appear to be especially sensitive to phonemes but do not modulate their processing of syllable stress. Thus, enhanced phonological processing in middle childhood appears to be restricted to those aspects of the speech signal that are relevant for acquiring an alphabetic writing system, namely phonemes, without generalizing to aspects of the speech signal that are not typically encoded in the writing system, namely prosody.

The second major finding of this study regards the independent processing of prosody and phonemes, as indicated by separate ERP phoneme priming and ERP stress priming. We uncovered that the main effects of stress overlap and the main effects of phoneme overlap did not interact in the first and third time window analyzed for the ERPs. Independent ERP phoneme priming and ERP stress priming in the same time windows provides evidence for two separate processing systems operating in parallel. This confirms the conclusion of independent processing of stress and phonemes that we have formerly drawn from ERPs recorded in cross-modal auditory-visual priming with adults (Friedrich et al., 2004).

Although ERPs allow only restricted conclusions about the localization of neuronal sources, different topographies of ERP phoneme priming and ERP stress priming support our conclusion of independent processing systems and are informative about the processing of stress. The left-lateralization of ERP phoneme priming replicates previous results obtained with unimodal auditory word onset priming (Friedrich et al., 2009; Schild et al., 2012) and cross-modal word onset priming (Friedrich et al., 2004, 2008; Friedrich, 2005). Bilateral stress priming replicates a previous result obtained with cross-modal auditory-visual word onset priming (Friedrich et al., 2004). The left-lateralization of phoneme priming is in line with the “asymmetric sampling in time” (AST) hypothesis stating that acoustic information varying on a small time-scale is processed predominantly in the left hemisphere (e.g., Poeppel, 2003; Poeppel et al., 2008). However, the AST hypothesis also states that the processing of acoustic information varying on a larger time-scale, such as syllable stress, is lateralized to the right hemisphere. This assumption is not confirmed by the present and previous bilateral ERP stress priming effects. Together our findings are in accordance with a meta-analysis of lesion literature revealing that linguistic prosodic perception is under bihemispheric control (Witteman et al., 2011).

Regarding behavioral stress priming, the present results obtained with a unimodal auditory paradigm can be integrated within previous work using a cross-modal priming paradigm. Similar to the former studies, we obtained the fastest responses for combined prime-target overlap in syllable stress and phonemes (see Soto-Faraco et al., 2001; Cooper et al., 2002; Friedrich et al., 2004; van Donselaar et al., 2005). This result reveals that pre-readers and readers rapidly integrate phonemes and prosody in ongoing spoken word recognition.

Most astonishingly, stress overlap without phoneme overlap elicited the slowest behavioral responses in the present study. This condition has been previously realized only in a single cross-modal priming study (Friedrich et al., 2004). There, behavioral responses for stress match were faster compared to stress mismatch. Here, we speculate that the enhanced response latencies for stress match in the present unimodal study result from a violation of basic rhythmic properties of speech in the stress match condition for initially stressed targets. In that condition, the stressed prime syllable is immediately followed by the stressed onset syllable of the target word. The juxtaposition of two stressed syllables, referred to as a “stress clash,” violates the regularly alternating sequence of stressed and unstressed syllables in continuous speech (Liberman and Prince, 1977; Tomlinson et al., 2013). The assumption that “stress clashes” delay the processing of stress-matching targets in unimodal priming has to be validated by adding initially unstressed targets to future designs.

ERP phoneme priming, as reflected in the auditory N100, in the P350 effect and in the central negativity, was largely comparable with the results of previous studies. Previously, enhanced left-lateralized negative-going amplitudes for phoneme match compared to phoneme mismatch have been obtained for adults in the N100 time window (100 to 300 ms; Friedrich et al., 2009; Schild et al., 2012), but not for children (Schild et al., 2011). Similarly, enhanced anterior positivity for phoneme mismatch has been obtained for adults and children in the P350 time window (300 to 400 ms). The bilateral distribution of the anterior P350 effect in the present study is integrated into a heterogeneous pattern of results regarding the lateralization of this ERP deflection, for which a bilateral distribution in adults (Schild et al., 2012) and pre-readers (Schild et al., 2011) has been obtained, in addition to a left-lateralized distribution in adults (Friedrich et al., 2009) and beginning readers (Schild et al., 2011).

The topography and polarity of amplitude differences characterizing ERP stress priming differed from ERP phoneme priming. Reversed to phoneme priming, the mean ERP amplitudes for stress match were more negative than the mean ERP amplitudes for stress mismatch starting at 200 ms after the target word onset. The bilateral posterior distribution relates ERP stress priming to N400-like central negativity and therewith to predictive phonological processing in unimodal auditory priming. Enhanced negativity for stress match compared to stress mismatch reflects that stress match is somewhat unexpected. Again, the atypical sequence of two stressed syllables in both stress match conditions might be relevant here. The stressed prime syllable followed by the stressed initial syllable of the target word violates the expectation of an alternating sequence of stressed and unstressed syllables in natural speech (Liberman and Prince, 1977). Enhanced N400 amplitudes for stress clash in a sentence context have been recently reported (Bohn et al., 2013). In other words, together with the behavioral priming results, we might interpret the enhanced central negativity for stress match as reflecting an unexpected stress clash.

Only between 300 and 400 ms was there an interaction between ERP phoneme priming and ERP stress priming. This interaction effect somewhat parallels the behavioral data. The condition that elicited the slowest responses, namely stress overlap without phoneme overlap (S+P−), also diverged in the P350 effect from the other conditions. Because a similar interaction was found over the anterior and posterior regions, we cannot unambiguously relate this event to either the P350 or the central negativity. However, a unifying interpretation of the data should focus on expectancy mechanisms. It appears that the target in the condition S+P− was the least expected, as the remaining three conditions were somehow still primed. S+P+ and S−P+ are primed by phoneme overlap with their preceding primes, whereas S−P− fulfills the expected pattern of alternating syllable stress between prime syllable and target syllable. This *post-hoc* interpretation must be examined further in future research.

In conclusion, we did not find different processing of syllable stress for pre-readers and readers in the present study. This contrasts to the evidence for enhanced and refined phoneme processing in readers that we found in the present study and in a former study (Schild et al., 2011). Thus, although developmental maturation and vocabulary growth might exert an influence on phonological processing throughout childhood (Walley et al., 2003) the present and previous results might be best explained by the influence of literacy. We conclude that literacy specifically improves the processing of those aspects of speech that find correlates in the written signal. Together these results converge to the conclusion of two separate processing streams for phonemes and prosody. ERPs point to functionally and anatomically distinct networks devoted to process both types of information. Age-related differences reveal that the processing of phonemes, but not the processing of prosody is modulated by literacy acquisition.

### Conflict of interest statement

The authors declare that the research was conducted in the absence of any commercial or financial relationships that could be construed as a potential conflict of interest.
